# Interferon-Gamma Release Assay Performance of Cerebrospinal Fluid and Peripheral Blood in Tuberculous Meningitis in China

**DOI:** 10.1155/2017/8198505

**Published:** 2017-02-20

**Authors:** Liping Pan, Fei Liu, Jinli Zhang, Xinting Yang, Shiqi Zheng, Jing Li, Hongyan Jia, Xiaoyou Chen, Mengqiu Gao, Zongde Zhang

**Affiliations:** ^1^Beijing Key Laboratory for Drug Resistant Tuberculosis Research, Beijing Chest Hospital, Capital Medical University, Beijing Tuberculosis and Thoracic Tumor Research Institute, 97 Machang Road, Tongzhou District, Beijing 101149, China; ^2^Tuberculosis Department, Beijing Chest Hospital, Capital Medical University, Beijing Tuberculosis and Thoracic Tumor Research Institute, 97 Machang Road, Tongzhou District, Beijing 101149, China; ^3^Neurology Department, People's Liberation Army 263 Hospital, 141 Yongshun South Street, Tongzhou District, Beijing 101149, China; ^4^Neurosurgery Department, Beijing Luhe Hospital, 82 Xinhua South Street, Tongzhou District, Beijing 101110, China

## Abstract

The aim of this study was to examine the performance of T-SPOT.TB on cerebrospinal fluid (CSF) and peripheral blood (PB) in diagnosis of tuberculous meningitis (TBM) in China. Of 100 patients with presumed TBM prospectively enrolled from Sep 2012 to Oct 2014, 53 were TBM (21 definite and 32 probable TBM cases) and 37 were non-TBM cases; the other 10 patients were excluded from analysis due to inconclusive diagnosis, no sufficient CSF samples, or incomplete follow-up. T-SPOT.TB on CSF and PB and routine laboratory tests of CSF were performed simultaneously. The receiver operating characteristic (ROC) curve and cut-off value of CSF T-SPOT.TB and routine CSF parameters were established between TBM and non-TBM group. The area under ROC curve (AUC) of the T-SPOT.TB on CSF and PB was 0.81 and 0.89, which was higher than that of the routine CSF parameters (AUC 0.67–0.77). Although the sensitivity of CSF T-SPOT.TB was lower than that of PB T-SPOT.TB (60.8% versus 90.6%, *P* < 0.001), the specificity of CSF T-SPOT.TB was significantly higher than that of PB T-SPOT.TB (97.2% versus 75.7%, *P* = 0.007). These results indicated that the diagnostic accuracies of PB and CSF T-SPOT.TB are higher than routine laboratory tests. Furthermore, the higher specificity of CSF T-SPOT.TB makes it a useful rule-in test in rapid diagnosis of TBM.

## 1. Introduction

Tuberculosis (TB) is one of the major infectious diseases threatening millions of lives worldwide, and 10.4 million new cases of TB are estimated by WHO in 2015 [1]. Tuberculous meningitis (TBM) is one of the most harmful TB. Although it accounts for 1% of all forms of TB, about 44–69% of TBM patients die despite antituberculosis chemotherapy, in developing countries [2]. Delays in diagnosis and treatment are regarded as major contributing factors in the high mortality reported in many recent series [3–5].

Until now, there is no definite laboratory test for early TBM diagnosis. It is diagnosed on the basis of clinical features, cerebrospinal fluid (CSF) studies, and radiological findings. Ziehl-Neelsen staining of CSF smears and CSF* Mycobacterium tuberculosis (M.TB)* culture are the definite methods for TBM diagnosis, but the smear has very low sensitivity (10–20%), while culture also lacks sensitivity and takes appropriately 6–8 weeks to obtain the result [6, 7]. The currently applied laboratory parameters including adenosine deaminase (ADA), lymphocyte count, glucose, and chloride concentration are of certain diagnostic value, but these parameters are frequently nonspecific. Both computed tomography (CT) and magnetic resonance images (MRI) are sensitive to the changes of TBM, particularly hydrocephalus and basal meningeal exudates, but these manifestations also lack specificity [8–10]. Therefore, an early, rapid, accurate diagnostic test is urgently needed for TBM diagnosis.

Recently, the interferon-gamma release assays (IGRAs) are being used increasingly to detect IFN-*γ* response of effector T cells to the* mycobacterium* tuberculosis-specific antigens, early secretary antigenic target 6 (ESAT-6), and culture filtrate protein 10 (CFP-10) [11]. This blood-based immunologic approach is suitable for the diagnosis of TB infection [12]. However, latent tuberculosis infection (LTBI) will inevitably affect the diagnostic accuracy of peripheral blood (PB) IGRAs. Thus the significance of this assay is questionable in high TB burden country. An alternative way that performs the IGRAs using the effector T cells at the infection site of disease may have higher interferon response frequency, compared with peripheral mononuclear cells (PBMC) [13, 14]. It has been reported that the IGRAs using body fluid manifest good diagnostic performance in extrapulmonary tuberculosis, such as tuberculous pleurisy [15, 16], suggesting that this assay may also have good performance in CSF. In recent years, a small number of studies have evaluated the T-SPOT.TB test on CSF for TBM diagnosis. However, the sample size of these studies was not large enough, and the sensitivity and specificity were controversial and varied in the range of 40–92% and 75–100% [17–19]. Furthermore, the study that evaluated the T-SPOT.TB test on CSF in high-burden setting, such as China, was limited.

To further determine whether the use of T-SPOT.TB test on CSF could be an accurate diagnostic method for TBM, we therefore conducted a prospective, blinded study to evaluate the performance of circulating and localized mononuclear cell-based enzyme-linked immunospot (ELISPOT) assays that included 100 subjects with suspected TBM in China.

## 2. Materials and Methods

### 2.1. Study Participants

A total of 100 inpatient subjects with suspected TBM were prospectively recruited between Sep 2012 and Oct 2014, from Beijing Chest Hospital, People's Liberation Army 263 Hospital, and Beijing Luhe Hospital. All included patients, or a direct relative for those with an abnormal mental state, gave informed consent to participate in the study. Medical records were collected on age, gender, underlying disease, and HIV serology status. Patients were tested with PB T-SPOT.TB and CSF T-SPOT.TB, and the following tests were also performed: routine clinical, microbiologic, histopathological, and biochemical examinations of CSF; and other samples were also performed, including routine chemistry, microscopy (Gram stain and for acid-fast bacilli),* M.TB* culture, TB polymerase chain reaction (PCR), bacterial and fungal culture, viral antibody, cryptococcal antigen latex agglutination test (CLAT), and CT/MRI images. The tuberculin skin test (TST) was not performed in these participants. Individuals were excluded if they had previous active tuberculosis history and tuberculosis contact history or they have received antituberculosis therapies before enrollment.

The study was performed in accordance with the guidelines of the Helsinki Declaration and its later amendments or comparable ethical standards and was approved by the Ethics Committee of the Beijing Chest Hospital, Capital Medical University.

### 2.2. Definitions and Diagnosis

The final diagnosis was based on clinical, histopathological, radiological, and microbiological information collected over at least 12 months of follow-up care. Finally, all of the patients were categorized as (1) definite TBM: the final diagnoses were made on the positive culture of* M.TB* from CSF,* M.TB* PCR assay, and the presence of caseating granuloma in meninges; (2) probable TBM: there were CSF findings of lymphocytic pleocytosis, increased protein levels and ADA, decreased glucose and chloride level, sterile cultures, and negative tests for other causes of meningitis, plus the following supporting criteria, which included CT and MRI revealing hydrocephalus, granulomas or basal exudates, evidence of extraneural TB and appropriate responses to antituberculous therapy; (3) non-TBM: an alternate definite cause for meningitis identified by microbiologic, histopathological, serologic examinations and response to appropriate nontuberculous therapy [20–22].

Throughout the study, the clinicians were blinded to the ELISPOT results, and the laboratory technicians were blinded to the diagnosis. Thus, laboratory interpretation and diagnosis were independent of the test results.

### 2.3. T-SPOT.TB Assay

The T-SPOT.TB test (Oxford Immunotec Ltd., Abingdon, UK) was performed according to the manufacturer's instructions. 6 ml of heparinized PB sample and 6 ml of CSF were collected. For CSF T-SPOT.TB, the specimens were centrifuged at 2,000 rpm for 10 min. The supernatants were discarded and the sediments were resuspended in 5 ml phosphate buffer; the subsequent processes were the same as those for the test using blood samples. This assay used 2.5 × 10^5^ PBMCs per well and 1 × 10^5^–2.5 × 10^5^ CSF mononuclear cells (CSFMC) per well. For the cell count in CSF was no more than 2.5 × 10^5^, we used the ratio between 2.5 × 10^5^, the target number, and the actual number to adjust the result. The procedure was performed in the plates precoated with anti-interferon-*γ* antibodies at 37°C for 16 to 20 hours. After application of alkaline phosphatase-conjugated second antibody and chromogenic substrate, the number of spot forming cells (SFCs) in each well was automatically counted with a CTL ELISPOT system (CTL-ImmunoSpot S5 Versa Analyzer, USA).

The optimal cut-off value of CSF T-SPOT.TB was derived by a receiver operating characteristic (ROC) curve according to SFCs between TBM and non-TBM. CSF T-SPOT.TB results were considered positive if ≥20 SFCs/million CSFMCs were counted after subtraction of the number of SFCs in the negative control well. PB T-SPOT.TB results were considered positive if ≥24 SFCs/million PBMCs were counted after subtraction of the number of SFCs in the negative control well or if the total number of SFCs was at least twice the number of SFCs in the negative control (according to the manufacturer). For both PB and CSF T-SPOT.TB, indeterminate results were defined (1) if the positive control failed; (2) the number of spots in the negative control well was more than 10; (3) if there was high background discoloration in the wells precluding meaningful evaluation of the plate.

### 2.4. Statistical Analysis

Continuous variables were compared using a *t*-test (for data with normal distribution) or Mann–Whitney *U* test (for data without normal distribution), as appropriate. A ROC curve was constructed by plotting the rate of sensitivity against the rate of (1 − specificity) results over a range of cut-off values of CSF T-SPOT.TB. Youden's Index was used to select the optimum cut points on the ROC curve (optimal balance between sensitivity and specificity). Diagnostic performance was expressed in terms of sensitivity, specificity, positive predictive value, negative predictive value, positive likelihood ratio, and negative likelihood ratio. Diagnostic accuracy was also assessed using the ROC curve. Chi-squared tests were used to compare categorical variables between TBM and non-TBM patients, definite TBM, and probable TBM. Significance was inferred for *P* < 0.05. All statistical analysis was performed using the commercial statistical software SPSS version 14.0 (SPSS, Inc., Chicago, IL, USA).

## 3. Results

### 3.1. Demographic and Clinical Characteristics

**Figure 1:**
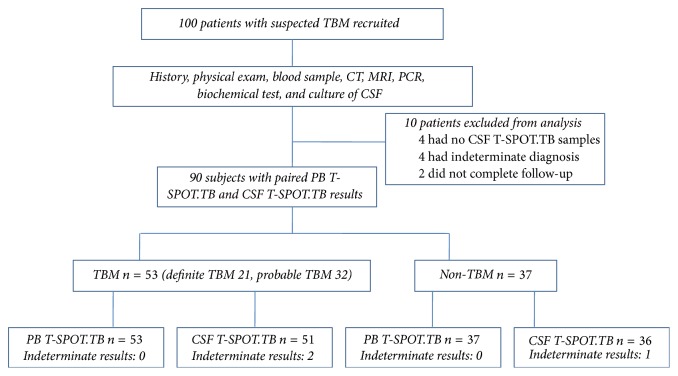
Flowchart of the study population. A total of 100 patients with suspected TBM were recruited; 90 were eligible to be included in the final analysis (these patients had paired PB T-SPOT.TB and CSF T-SPOT.TB).

Among the 100 patients with suspected TBM (Figure 1), 10 patients were excluded from the study, of which 4 had no sufficient CSF T-SPOT.TB samples, 4 had inconclusive diagnosis, and 2 did not complete the follow-up. The remaining 53 were diagnosed as TBM and 37 were diagnosed as non-TBM cases. The TBM group consisted of definite TBM group (*n* = 21) and probable TBM group (*n* = 32). Among these definite TBM patients, 15 were confirmed with* M.TB* PCR, 5 were confirmed with* M.TB* culture, and 1 was confirmed with meningeal pathology. Major clinical characteristics of the 90 recruited subjects were summarized in Table 1. Two patients in the study were positive in HIV serology test; these two patients were diagnosed as having cryptococcal meningitis (CM). The alternate diagnoses in the non-TBM group included viral meningitis, acute bacterial meningitis, CM, meningeal malignant tumor, and other CNS diseases. In some TBM patients, combined with extraneural tuberculosis, the distribution of other affected organs was highly heterogeneous which involved lung, bone/joint, kidney, lymph node, and liver/spleen. Total white blood cell (WBC) count, lymphocyte count, ADA, and protein level of CSF in TBM group were higher than those in non-TBM group, while the glucose level, chloride concentration of CSF, and CSF/serum glucose ratio in TBM group were lower than those in non-TBM group. Five patients within the TBM group and two patients within the non-TBM group died in the follow-up period.

### 3.2. Establishment of Receiver Operating Characteristic (ROC) Curve of CSF and PB

**Figure 2:**
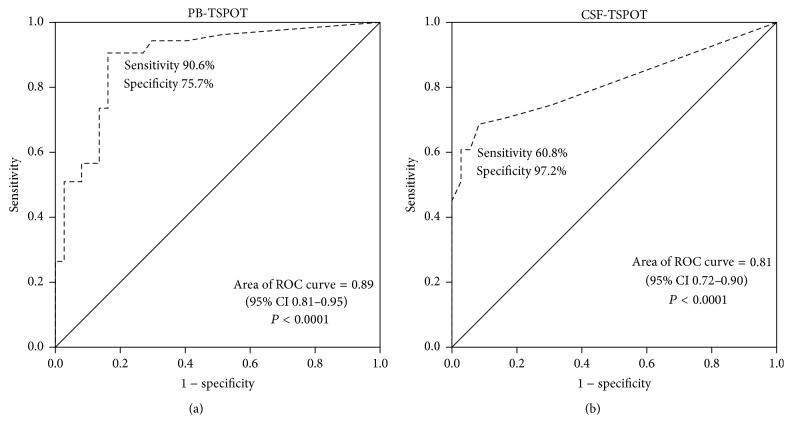
Receiver operating characteristic (ROC) curve in PB T-SPOT.TB (a) and CSF T-SPOT.TB (b) for the diagnosis of tuberculous meningitis.

Compared to PB, distinct cut points in specific body compartments have been utilized. However, no cut-off for CSF, which has a unique physiologic and anatomic characteristic, has been defined. We established a new ROC curve of CSF between TBM and non-TBM group and defined 20 SFCs per million mononuclear cells as the optimal cut-off value, considering the higher specificity at the expense of sensitivity. The cut-off value of ≥24 SFCs/million PBMCs was used for the PB T-SPOT.TB by manufacturer recommendation. On the basis of this analysis, the area under ROC curve (AUC) of CSF T-SPOT.TB and PB T-SPOT.TB was 0.81 (95% CI 0.72–0.90) and 0.89 (95% CI 0.81–0.95), respectively (Figure 2, Table 2). The definite TBM and probable TBM were also analyzed separately (Table 2). The AUC of CSF T-SPOT.TB and PB T-SPOT.TB in patients with definite TBM versus non-TBM was 0.80 (95% CI 0.67–0.89) and 0.93 (95% CI 0.83–0.98), respectively, while the AUC of CSF T-SPOT.TB and PB T-SPOT.TB in patients with probable TBM versus non-TBM was 0.82 (95% CI 0.71–0.91) and 0.87 (95% CI 0.76–0.94), respectively.

### 3.3. Diagnostic Performance of T-SPOT.TB in CSF and PB

**Figure 3:**
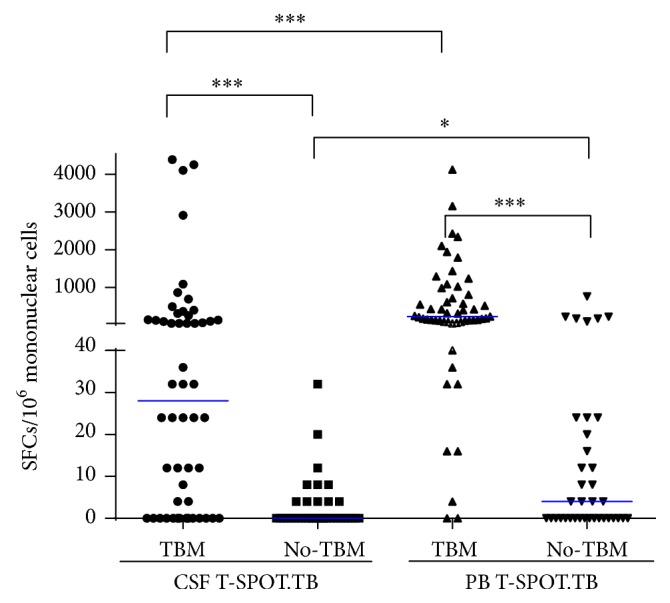
Scatter plot of spot forming cells using PB T-SPOT.TB and CSF T-SPOT.TB between tuberculous meningitis and no tuberculous meningitis. Group comparison is carried out using Mann–Whitney *U* test. ^*∗*^*P* < 0.05; ^*∗∗∗*^*P* < 0.0001.

Compared with the non-TBM group, the TBM group had significantly more SFCs in CSF T-SPOT.TB [32 (0–142)/10^6^ PBMCs versus 0 (0–4)/10^6^ PBMC, *P* < 0.0001] and PB T-SPOT.TB [224 (86–892)/10^6^ PBMCs versus 4 (0–22)/10^6^ PBMC, *P* < 0.0001]. Within the TBM group, there were more SFCs in PB T-SPOT.TB [224 (86–892)/10^6^ PBMCs] than those in CSF T-SPOT.TB [32 (0–142)/10^6^ CSFMCs] (*P* < 0.0001) (Figure 3). In terms of CSF T-SPOT.TB results, indeterminate rate was 3.3% (3/90; 95% CI 0.1%–9.4%). There were 2 indeterminate results in TBM group and 1 indeterminate result in non-TBM group, respectively. All indeterminate results were due to failed positive control wells. There was no indeterminate result in PB T-SPOT.TB. The diagnostic performance of CSF T-SPOT.TB and PB T-SPOT.TB for 90 subjects was presented in Table 2. The overall sensitivity of PB T-SPOT.TB was higher (90.6%, 95% CI 79.3%–96.9%) than that of CSF T-SPOT.TB (60.8%, 95% CI 46.1%–74.2%) (*P* < 0.001), while the specificity of CSF T-SPOT.TB (97.2%, 95% CI 85.5%–99.9%) was significantly higher than that of PB T-SPOT.TB (75.7%, 95% CI 58.8%–88.2%) (*P* = 0.007). Compared with the performance of T-SPOT.TB on CSF or PB alone, the combination of T-SPOT.TB on CSF and PB was also evaluated. However, neither the double positive of CSF and PB T-SPOT.TB nor the single positive of CSF or PB T-SPOT.TB presented appearing advantages. The CSFMCs/PBMCs ratio was also calculated in TBM and non-TBM group. All of the non-TBM patients presented CSFMCs/PBMCs ratio < 1.0, but CSFMCs/PBMCs ratio > 1.0 was found in only 8 cases of 53 TBM patients, which showed a higher specificity and lower sensitivity.

**Figure 4:**
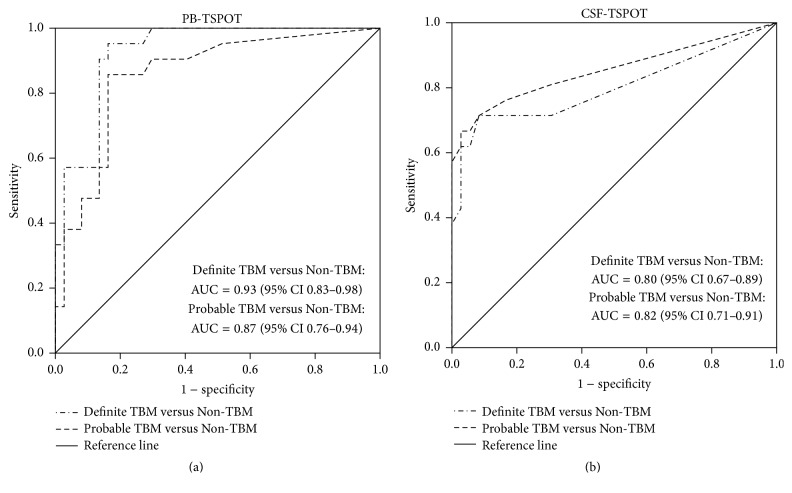
Diagnostic performance of PB T-SPOT.TB (a) and CSF T-SPOT.TB (b) between definite tuberculous meningitis and probable tuberculous meningitis. AUC, area under ROC curve.

**Figure 5:**
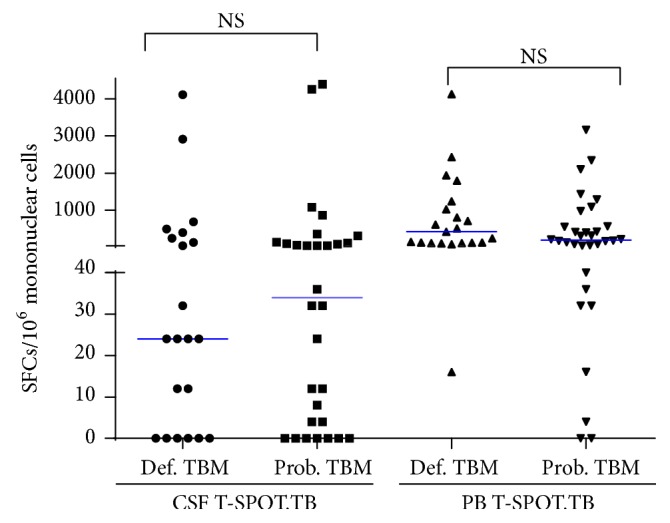
Scatter plot of spot forming cells using PB T-SPOT.TB and CSF T-SPOT.TB between definite tuberculous meningitis and probable tuberculous meningitis. Group comparison is carried out using Mann–Whitney *U* test. NS: not significant.

As shown in Table 2, the sensitivity of CSF T-SPOT.TB and PB T-SPOT.TB in patients with definite TBM compared with non-TBM was 61.9% (95% CI 40.9%–79.3%) and 95.2% (95% CI 77.3%–99.2%), respectively. The probable TBM was also compared with non-TBM; the sensitivity of CSF T-SPOT.TB and PB T-SPOT.TB was 60.0% (95% CI 42.3%–75.4%) and 87.5% (95% CI 71.9%–95.0%), respectively. There was no significant difference in diagnostic performance of PB T-SPOT.TB between definite TBM and probable TBM (Figure 4(a)). Also, there was no significant difference in diagnostic performance of CSF T-SPOT.TB between these two subgroups (Figure 4(b)). Furthermore, there was no significant difference in SFCs of CSF T-SPOT.TB and PB T-SPOT.TB between definite TBM and probable TBM subgroups (Figure 5).

### 3.4. Diagnostic Performance of the Routine Laboratory Parameters of CSF

In the routine laboratory tests of CSF, the cut-off values of total WBC count, protein level, lymphocyte count, ADA, glucose, chloride concentration, and the CSF/serum glucose ratios were established by the ROC curves between TBM and non-TBM group. The AUC of total WBCs, lymphocytes, ADA, CSF/serum glucose ratios, CSF glucose, CSF protein levels, and CSF chloride concentration were 0.67, 0.70, 0.68, 0.75, 0.77, 0.72, and 0.68, respectively (Table 3). And the diagnostic accuracy of total WBCs, lymphocytes, ADA, CSF/serum glucose ratios, CSF glucose, CSF protein levels, and CSF chloride concentration were 61.1% (55/90), 73.3% (66/90), 66.7% (60/90), 73.3% (66/90), 77.8% (70/90), 76.7% (69/90), and 65.6% (59/90).

## 4. Discussion

The sensitivity of PB T-SPOT.TB (90.6%) for detecting TBM was in line with previous studies conducted in pulmonary and extrapulmonary TB [23, 24]. The lower specificity (75.7%) might be due to the high latent TB infection (LTBI) in the non-TBM group, because the PB T-SPOT.TB cannot discriminate active TB and LTBI. The rate of positive PB T-SPOT.*TB* results in the non-TBM group here being consistent with the prevalence of LTBI in China, which has been investigated using IGRA assays and ranged from 19.0% to 33.6% among different population groups [25–28].

Up to date, there is no definite cut-off value for CSF T-SPOT.TB test. Considering the conventional laboratory tests of CSF all lack specificity and the false positive results may lead to inappropriate antituberculous therapy and unnecessary pain (the antituberculous therapy for TBM was recommended for at least 9 to 12 months), we sought to improve the specificity for the diagnosis of TBM at the expense of the sensitivity in the present study. More than 20 SFCs/10^6^ CSFMCs were selected as the cut-off value for TBM diagnosis, and this cut-off value was also used in the previous study [17].

Based on this cut-off value, a relatively high specificity of CSF T-SPOT.TB test was yielded and it was significantly higher than that of PB T-SPOT.TB, which indicated that CSF T-SPOT.TB could be a rapid rule-in test for TBM diagnosis. These results were consistent with previous studies either using the manufacturer recommended cut-off value or self-constructed cut-off values for CSF T-SPOT.TB. Furthermore, these results were also similar to the findings when using pleural fluid or bronchoalveolar lavage fluid [15, 29]. The higher specificity of body fluid T-SPOT.TB was possibly due to the compartmentalization of antigen-specific effector T cells.* M.TB*-specific effector T cells could be recruited to the infection site in case of active TB, and then the enumeration of effector T cells by T-SPOT.TB at the infection site could present a higher specificity of TB diagnosis in comparison with that obtained from blood assay [30, 31]. However, the sensitivity and the SFCs of CSF T-SPOT.TB were much lower than those of PB T-SPOT.TB. These results were in line with previous findings for TBM [19, 30] but are lower than those on pleural fluid or bronchoalveolar lavage fluid [15, 29]. We assumed that the possible reason of lower sensitivity and enumeration of antigen-specific T cells could be the protective effect of blood-brain barrier (BBB). Although TB-inflamed BBB with increased permeability allow some lymphocytes migration, the number of lymphocytes (including* M.TB* specific T cells) in subarachnoid cavity was far lower than that in bronchus and pleural cavity [29, 32]. Nevertheless, although the sensitivity of CSF T-SPOT.TB was lower on the basis of this cut-off value, it was still higher than the microbiological tests in our study. Of the 53 TBM patients, only 15 were* M.TB* PCR positive (28.3%) and 5 were culture-positive (9.4%); no one was AFB-positive.

Between definite TBM and probable TBM subgroups, there was no significant difference in SFCs and overall diagnostic performance of CSF T-SPOT.TB and PB T-SPOT.TB. The final diagnoses of definite TBM were mainly made on the positive culture of* M.TB* and* M.TB* PCR from CSF. Theoretically, the bacterial load of definite TBM was higher than that of probable TBM. However, the* M.TB* antigens-specific IFN-*γ* responses of PB and CSF were similar in these two subgroups. These results indicated that IFN-*γ* response may be not only correlated with the bacterial load of human body, but also associated with the host responsiveness to these antigens and the extent of host-pathogen interactions [33]. Furthermore, these results may also suggest that the probable TBM cases recruited in our study were the true TBM, although they lack the microbiological confirmation.

Our study also indicated that the routine laboratory tests of CSF had poor value for the diagnosis of TBM. These data were consistent with other recent researches [17, 30]. CSF ADA presented a relatively higher specificity (94.6%), but the sensitivity was decreased (47.2%). In comparison with these CSF parameters, PB and CSF T-SPOT.TB had a relatively higher diagnostic accuracy for TBM diagnosis. The ROC analysis showed a balanced sensitivity (66.7%) and specificity (86.1%) of CSFMC/PBMC ratio in diagnosis of TBM, when using the 0.02 as cut-off value. However, previous studies have generally indicated that use of a higher ratio of CSFMC/PBMC T-SPOT.TB (≥1 or ≥2) could present a good specificity in TBM diagnosis [18, 30, 31]. Similar to these studies, we also found that all of the non-TBM patients in our study presented CSFMCs/PBMCs ratio < 1.0, which resulted in a specificity of 100%, but the sensitivity was decreased to 15.1% (8/53).

The limitations of our study need to be addressed. First, the number of enrolled cases was not large enough, due to the lower incidence of TBM in our country and the difficulties in definite diagnosis of TBM. The majority of patients were probable TBM cases that were diagnosed mainly by routine CSF tests, CT/MRI findings, evidence of extraneural TB, and appropriate responses to anti-TB chemotherapy. However, no significant difference was detected between definite TBM and probable TBM; thus inclusion of those uncertain bacteriological cases may not cause bias in the performance for T-SPOT.TB. Second, the CSF samples from 3 of the 90 (3.3%) participating subjects yielded indeterminate T-SPOT.TB results, and all these indeterminate results were due to failed positive control wells. The possible cause of indeterminate CSF T-SPOT.TB may be due to the insufficient volume of CSF and subsequently limiting number of CSF lymphocytes, although 6 ml CSF were collected from each subject under ethical consideration. Third, there are only five patients with CM in non-TBM group in our cohort. Among them, one patient has positive PB T-SPOT.TB and CSF T-SPOT.TB results. Although the incidence of CM was obviously lower than that of TB in low HIV burden country, the mortality of patients with CM is higher than those with TBM. Differential diagnosis between TBM from CM is difficult by current laboratory tests [20]. Therefore, further researches on this issue should recruit more patients with CM and validate the differential value of T-SPOT.TB between TBM and CM. Finally, since there were only 2 patients who were serum-positive for HIV in our study, the results presented here only apply to the low HIV prevalence settings; further research in the high TB and HIV coinfection burden settings should be conducted to evaluate the performance of CSF T-SPOT.TB for TBM diagnosis.

## 5. Conclusion

In conclusion, these results indicated that the diagnostic accuracies of PB and CSF T-SPOT.TB are higher than routine laboratory tests. Furthermore, the higher specificity of CSF T-SPOT.TB makes it a useful rule-in test in rapid diagnosis of tuberculous meningitis. However, further prospective studies with larger sample size will be needed to validate the practical use of this CSF immunological assay in high TB burden country.

## Figures and Tables

**Table 1:** Clinical characteristics in study population (*n* = 90).

Characteristics	TBM group (*n* = 53)	Non-TBM group (*n* = 37)	*P* value
Age, median (IQR), yrs	31 (23–45)	27 (23–47)	0.938
Gender (female/male)	19/34	9/28	0.245
HIV infection	0	2	0.087
Cause of diseases			
Tuberculous meningitis	53	NA	NA
Viral meningitis	NA	22	NA
Purulent meningitis	NA	4	NA
Cryptococcal meningitis	NA	4	NA
Meningeal malignant tumor	NA	2	NA
Subarachnoid hemorrhage	NA	1	NA
Other causes	NA	4	NA
Underlying diseases			
Diabetes mellitus	4	3	0.922
Viral hepatitis	2	2	0.712
SLE	1	0	0.414
Myeloma	1	0	0.414
CSF profile			
Total WBC count, median cell ×10^3^/ml (IQR)	54 (20–116)	15 (6.5–72)	0.006
Lymphocyte count, median cell ×10^3^/ml (IQR)	45 (19–80)	12 (5.5–63)	0.002
ADA, median U/L (IQR)	4.1 (1.9–6.0)	2.2 (1.75–3.0)	0.005
CSF/serum glucose ratio, median ratio (IQR)	0.45 (0.38–0.56)	0.64 (0.50–0.70)	<0.001
Glucose, median mmol/L (IQR)	2.20 (1.73–2.75)	3.10 (2.80–3.65)	<0.001
Protein level, median g/L (IQR)	0.75 (0.63–1.10)	0.40 (0.30–0.90)	0.001
Chloride concentration, median mmol/L (IQR)	117.1 (112.6–121.0)	120.1 (118.5–122.0)	0.003

*Note*. NA: not applicable; IQR: interquartile range.

**Table 2:** Diagnostic performance of PB T-SPOT.TB and CSF T-SPOT.TB in the study population (*n* = 90).

	Cut point	Sensitivity	Specificity	PPV	NPV	LR+	LR−	AUC
*Definite and probable TBM *(*n* = 53)* compared with non-TBM *(*n* = 37)								
PB T-SPOT.TB/10^6^ PBMC	≥24	90.6^a^ (79.3–96.9)	75.7^b^ (58.8–88.2)	84.2 (72.1–92.5)	84.8 (68.1–94.9)	3.72 (2.10–6.60)	0.12 (0.05–0.30)	0.89 (0.81–0.95)
CSF T-SPOT.TB/10^6^CSFMC^*∗*^	≥20^#^	60.8^a^ (46.1–74.2)	97.2^b^ (85.5–99.9)	96.9 (83.5–99.9)	63.6 (49.6–76.2)	21.88 (3.10–153.10)	0.40 (0.30–0.60)	0.81 (0.72–0.90)
CSF/PB SFCs ratio	≥0.02^#^	66.7 (52.1–79.2)	86.1 (70.5–95.3)	87.2 (72.6–95.7)	64.6 (49.5–77.8)	4.80 (2.10–11.10)	0.39 (0.30–0.60)	0.77 (0.67–0.86)
CSF and PB T-SPOT.TB positive	—	58.8 (44.2–72.4)	97.2 (85.5–99.9)	96.8 (82.9–99.9)	62.5 (48.5–75.1)	21.18 (3.00–148.30)	0.42 (0.30–0.60)	0.78 (0.68–0.86)
CSF or PB T-SPOT.TB positive	—	92.5 (81.8–97.9)	75.0 (57.8–87.9)	84.5 (72.6–92.7)	87.1 (69.8–96.5)	3.70 (2.10–6.50)	0.10 (0.04–0.30)	0.84 (0.74–0.91)
Definite TBM (*n* = 21) compared with non-TBM (*n* = 37)								
PB T-SPOT.TB/10^6^ PBMC	≥24	95.2^c^ (77.3–99.2)	83.8 (68.0–93.8)	76.9 (55.9–91.2)	96.9 (83.8–99.9)	5.87 (2.80–12.30)	0.06 (0.01–0.40)	0.93 (0.83–0.98)
CSF T-SPOT.TB/10^6^ CSFMC	≥20	61.9^d^ (40.9–79.3)	97.2 (85.5–99.9)	92.9 (66.1–99.8)	81.4 (66.4–91.7)	22.29 (3.10–158.50)	0.39 (0.20–0.70)	0.80 (0.67–0.89)
CSF/PB SFCs ratio	≥0.02	61.9 (38.4–81.9)	86.1 (70.5–95.3)	72.2 (46.5–90.3)	79.5 (63.5–90.7)	4.46 (1.80–10.70)	0.44 (0.30–0.80)	0.76 (0.63–0.87)
Probable TBM (*n* = 32) compared with non-TBM (*n* = 37)								
PB T-SPOT.TB/10^6^ PBMC	≥24	87.5^c^ (71.9–95.0)	83.8 (68.0–93.8)	82.4 (65.5–93.2)	88.6 (73.3–96.8)	5.40 (2.60–11.40)	0.15 (0.06–0.40)	0.87 (0.76–0.94)
CSF T-SPOT.TB/10^6^CSFMC	≥20	60.0^d^ (42.3–75.4)	97.2 (85.5–99.9)	94.7 (74.0–99.9)	74.5 (59.7–86.1)	21.6 (3.10–152.50)	0.41 (0.30–0.60)	0.82 (0.71–0.91)
CSF/PB SFCs ratio	≥0.02	70.0 (50.6–85.3)	86.1 (70.5–95.3)	80.8 (60.6–93.4)	77.5 (61.5–89.2)	5.04 (2.20–11.80)	0.35 (0.20–0.60)	0.78 (0.66–0.87)

PBMC, peripheral blood mononuclear cells; CSF, cerebrospinal fluid; CSFMC, cerebrospinal fluid mononuclear cells; SFCs, spot forming cells; PPV, positive predictive value; NPV, negative predictive value; LR+, positive likelihood ratio; LR−, negative likelihood ratio; AUC, area under ROC curve.

^#^The cut-off of the parameters was established by a new ROC curve between TBM and non-TBM group.

^*∗*^There were 2 and 1 indeterminate CSF T-SPOT.TB results in TBM group and non-TBM group, respectively.

^a^*P* < 0.001, comparison of PB T-SPOT.TB and CSF T-SPOT.TB in TBM group; ^b^*P* = 0.007, comparison of PB T-SPOT.TB and CSF T-SPOT.TB in non-TBM group.

^c^*P* = 0.064, sensitivity comparison of PB T-SPOT.TB between definite TBM and probable TBM group.

^d^*P* = 0.891, sensitivity comparison of CSF T-SPOT.TB between definite TBM and probable TBM group.

**Table 3:** Diagnostic performance of routine laboratory parameters of CSF in the study population (*n* = 90).

	Cut point^#^	Sensitivity	Specificity	PPV	NPV	LR+	LR−	AUC
Total WBC count, cell ×10^3^/ml	≥46	54.7 (40.4–68.4)	70.3 (53.0–84.1)	72.5 (56.1–85.4)	52.0 (37.4–66.3)	1.84 (1.10–3.20)	0.64 (0.40–0.90)	0.67 (0.56–0.77)
CSF lymphocytes, cell ×10^3^/ml	≥16	79.3 (65.9–89.2)	64.9 (47.5–79.8)	76.4 (63.0–86.8)	68.6 (50.7–83.1)	2.26 (1.40–3.60)	0.32 (0.20–0.60)	0.70 (0.59–0.79)
CSF ADA, U/L	≥4.7	47.2 (33.3–61.4)	94.6 (81.8–99.3)	92.6 (75.7–99.1)	55.6 (42.5–68.1)	8.73 (2.20–34.60)	0.56 (0.40–0.70)	0.68 (0.57–0.77)
CSF/serum glucose ratio	≤0.56	76.0 (61.8–86.9)	69.7 (51.3–84.4)	79.2 (65.0–89.5)	65.7 (47.8–80.9)	2.51 (1.50–4.30)	0.34 (0.20–0.60)	0.75 (0.64–0.83)
CSF glucose, mmol/L	≤2.8	81.1 (68.0–90.6)	73.0 (55.9–86.2)	81.1 (68.0–90.6)	73.0 (55.9–86.2)	3.00 (1.70–5.20)	0.26 (0.10–0.50)	0.77 (0.67–0.85)
CSF protein level, g/L	≥0.6	83.0 (70.2–91.9)	67.6 (52.2–82.0)	78.6 (65.6–88.4)	73.5 (55.3–87.3)	2.56 (1.60–4.10)	0.25 (0.10–0.50)	0.72 (0.61–0.81)
CSF chloride concentration, mmol/L	≤117.7	52.8 (38.6–66.7)	83.8 (68.0–93.8)	82.4 (65.5–93.2)	55.4 (41.5–68.7)	3.26 (1.50–7.10)	0.56 (0.40–0.80)	0.68 (0.58–0.78)

CSF, cerebrospinal fluid; ADA, adenosine deaminase; WBC, white blood cell; PPV, positive predictive value; NPV, negative predictive value; LR+, positive likelihood ratio; LR−, negative likelihood ratio; AUC, area under ROC curve.

^#^The cut-off of the parameters was established by a new ROC curve between TBM and non-TBM group.
